# Y-chromosomal Status of Six Indo-European-speaking Arab Subpopulations in Chaharmahal and Bakhtiari Province, Iran

**Published:** 2018-03

**Authors:** Elham BANIMEHDI-DEHKORDI, Behnaz SAFFAR, Mostafa SHAKHSI-NIAEI

**Affiliations:** Dept. of Genetics, Faculty of Science, Shahrekord University, Shahrekord, Iran

**Keywords:** Arab subpopulation, Haplogroup, Polymorphism, Iran, Y-chromosome

## Abstract

**Background:**

We analyzed the Y-chromosome haplogroups of six documented Arab subpopulations that accommodated separately in different counties of Chaharmahal and Bakhtiari Province but nowadays speak Indo-European language (Luri and Farsi).

**Methods:**

This was an outcome study conducted in 2015 to test whether there was any genetic relatedness among some Indo-European-speaking Arab subpopulation accommodated in a geographically similar region, Chaharmahal and Bakhtiari Province, Iran. Seven main Y-chromosome bi-allelic markers were genotyped in six documented Arab subpopulations. Therefore, after DNA extraction from blood samples, PCR reaction carried out by designed primers for J1-M267, J2-M172, and J-M304, I-M170, IJ-M429, F-M89 and K-M9 markers. Then PCR products after quality control on agarose gel were sequenced.

**Results:**

Most subjects (83.3%) belonged to F-M89 haplogroup. These subjects belonged to K-M9 (40%), J2-M172 (40%) and I-M170 (20%). Generally, there were at least three genetically distinct ancestors with a divergence date of about 22200 yr for I, 429000 for J and 47400 before present for K haplogroup and may show separate historical migrations of studied populations. As the most recent common ancestor (MRCA) of most of these populations, haplogroup F, lived about 40000–50000 yr ago, the data do not support a nearly close genetic relationship among all of these populations. However, there were populations with same haplogroups J2 (n=2), K (n=2), or with a closer MRCA, IJ haplogroups, among I and J2 haplogroups. Finding haplogroup I, a specific European haplogroup, among Arab populations was not expected.

**Conclusion:**

Identification of various haplogroups in Arab subpopulations despite its small area and geographically conserved region of this part of Iranian plateau was unexpected.

## Introduction

Correlation of genetic and linguistic relationships of populations may confirm deep populational history. Albeit, this kind of correlation can be disturbed by geography, as may geographically adjacent but genetically distinct populations speak related languages. Therefore, genetical analysis of groups whose geographic neighbors spoke originally different languages looks necessary to check their populational history (1).

A correlation of speaking languages and genetical background of neighboring groups have been analyzed and different results have been achieved (2–8). For example, there was geographical, and genetical proximity but different spoken languages (2–4), in some other studies there were distinct geographical groups but showed similar language, and genetic background (5,6) and there are other studies among similar geographical areas, and languages detected different genetical backgrounds (7,8).

In studies of ancient human migration and population genetics, possible time of origin of Y-chromosome haplogroup markers are estimated and widely used. For example, possible time of origin of about 22200 yr before present (YBP) for haplogroup I-M170 (9), 42900 YBP for JM304 (10), 47400 YBP for K-M9 (11) and about 40000–50000 YBP for F haplogroup (12) have been reported.

Some researchers used Y-chromosome haplogroups to elucidate paternal connections in Iranian populations. For example, diversity of Iranian Azeri’s Y-Chromosomal haplogroups among Turkish-Speaking Populations of the Middle East was investigated. They could detect J, BR*, P*, E* and R1a1 Y-chromosomal haplogroups as frequent haplogroups (between 10%–40%) in North West of Iran (6). New clues from the Y-Chromosome Variation of Modern Iranians were reported and found different haplogroups which J2, R, G, J1, Q and L haplogroups showed higher frequency (13).

Chaharmahal and Bakhtiari Province is located in Southwest region of conserved Iranian plateau. It is surrounded by Khuzestan, Isfahan and Lorestan, Kohgiluyeh and Boyer-Ahmad provinces. It belongs to provinces having high frequency of Arab people because of geographically vicinity to Arabic countries. However, in this province, Bakhtiari tribe is dominant population with Luri speaking language.

As there are very few studies on Y-chromosome haplogroups in Iranian populations and also no available data about haplogroup situation of Arab people in Chaharmahal and Bakhtiari Province, in this study we analyzed the Y-chromosome haplogroups of six documented Arab subpopulations which accommodated separately in different counties of Chaharmahal and Bakhtiari Province but nowadays speak Indo-European language (Luri and Farsi).

## Materials and Methods

**Fig. 1:**
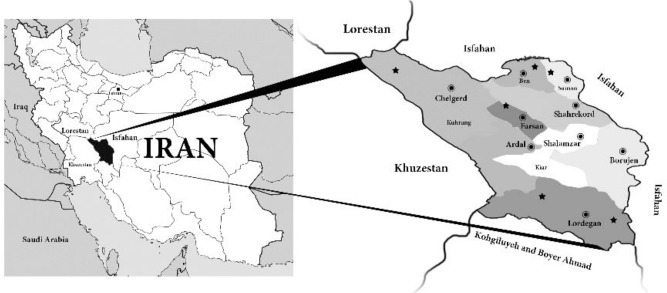
Main documented locations of conserved Arab subpopulations in Chaharmahal and Bakhtiari province. Black asterisks show the six considered locations.

After inquiries in 2015 from endowments organization of Chaharmahal and Bakhtiari Province as well as checking other related documents (14), collectively 95 places with a sign of Arab attendance (also called Imamzadeh) were recognized throughout the province. However, only six places still contained their available attributed descendants which are as follows: Imamzadeh Isa, Sar-Agha-Sayyed historical village, Koohrang county; Imamzadeh Ahmad, Baba-Heidar City, Farsan county; Imamzadeh Abdorrahman-Bideleh, Bideleh village, Lordegan county; Imamzadeh Mirahmad-Shirmard, Shirmard village, Lordegan county; Imamzadeh Sayyed-Bahaoddin-Muhammad, Sheikh Shaban village, Ben county; and Imamzadeh Baba Pirahmad in Ben, Ben county (14). The locations of these six places are shown in Fig. 1. All of these Arab subpopulations speak Luri except for those live in Sheikh Shaban and Ben, who speak Farsi but with an Arabic pronunciation.

From each of six different but patrilineally-conserved subpopulations, a volunteer enrolled as a representative of that subpopulation and his validated pedigree was checked. For each person, blood sample was collected in disposable sampling tubes containing EDTA and transferred to the genetics laboratory of the faculty of sciences, Shahrekord University. The DNA contents were extracted using Cinnagen DNA extraction kit and the DNA qualities were checked by electrophoresis on the 1% agarose gel.

The primers were designed using Primer3Input v.0.6 tool and primer pairs were checked to be site-specific using Primer-Blast tool (Table 1), for following haplogroup markers: F-M89, IJ-M429, K-M9, J1-M267, J-M304, J2-M172, and IM170 (Table 2) (YDNASNPIdex, 2014, http://www.isogg.org/tree/ISOGG_YDNA_SNP_Index14.html).

**Table 1:** Characteristics of designed primers for evaluation of haplogroups

***Primer***	***5′-3′ sequence***	***Primer length***	***Product size***
HG F-Forward	AGAAGCAGATTGATGTCCCAC	21	595
HG F-Reverse	GGAAGTGGTGAGCGAATGT	19	
HG IJ-Forward	AGGAGGAGGATGAAGCAGAG	20	468
HG IJ-Reverse	ATCACAAACTGCCCTCCAAT	20	
HG K-Forward	GCAGCATATAAAACTTTCAG	20	340
HG K-Reverse	AAAACCTAACTTTGCTCAAG	20	
HG J1-Forward	CATTATCCTGAGCCGTTGTC	20	730
HG J1-Reverse	AAAGCAAGTGGCCCAATAA	19	
HG J2-Forward	GGCCAGCTTTGTGCATTT	18	751
HG J2-Reverse	ACTGCATTAGCCACATTTGC	20	
HG J-Forward	ACTGTGCTTGCCTTTTGTG	19	748
HG J-Reverse	TGTTGCCTCTGCTTAAATGA	20	
HG I-Forward	TGCTTCACACAAATGCGT	18	399
HG I-Reverse	ACTTTCAACATTTAAGACC	19	

**Table 2:** The characteristic of investigated haplogroups in this research (Y-DNASNPIndex, 2014)

***Haplogroup***	***SNP***	***Ref SNP ID***	***Y-position***	***Mutation***
F	M89	rs2032652	21917313	C>T
K	M9	rs3900	21730257	C>G
IJ	M429	rs17306671	14031334	T>A
I	M170	rs2032597	14847792	A>C
J	M304	rs13447352	22749853	A>C
J1	M267	rs9341313	22741818	T>G
J2	M172	rs2032604	14969634	T>G

Related regions were amplified by PCR method and PCR products were sent for sequencing to MacroGene Company, Korea. Sequencing results were analyzed for existence of each haplogroup allele using Sequencher software v.5.2.4.

### Ethics

The Ethics Committee of Shahrekord University (No.161/133) approved this study. Participation was voluntary and linked to the signing of the Informed Consent Form.

## Results

**Fig. 2:**
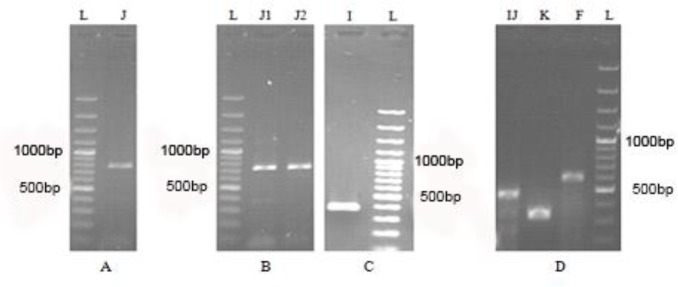
Electrophoresis of the PCR products of examined markers in attributed individuals to one of Imamzadehs in Chaharmahal and Bakhtiari Province. L: Ladder 100 bp (SM0321). Expected sizes of related PCR products for analysis of different haplogroup markers (as written on top of the pictures) are as follows: F-M89: 595 bp, IJ-M429:468 bp, K-M9:340 bp, I-M170: 399 bp, JM304: 784 bp, J1-M267: 730 bp and J2-M172: 751 bp.

Results of electrophoresis of the PCR products related to different haplogroup markers are shown in the Fig. 2.

Results of sequence analysis for each haplogroup marker are seen in Table 3. Five out of six subpopulations belonged to F-M89 haplogroup and only one of them did not show the F marker polymorphism and therefore, could be one of upper branches of Y-chromosome phylogenic tree like DE haplogroup. Among F-M89 subpopulations, haplogroups of K-M9 (40%), J2-M172 (40%) and I-M170 (20%) were observed.

**Table 3:** Haplogroup analysis of studied Arab subpopulations in Chaharmahal and Bakhtiari Province, Iran

***Sample***	***F-M89***	***K-M9***	***IJ-M429 J***	***-M304***	***J1-M267***	***J2-M172***	***I-M170***
	***C>T***	***C>G***	***T>A***	***A>C***	***T>G***	***T>G***	***A>C***
1	T	C	A	C	T	G	A
2	T	C	A	C	T	G	A
3	T	C	A	A	T	T	C
4	C	C	T	A	T	T	A
5	T	G	T	A	T	T	A
6	T	G	T	A	T	T	A

## Discussion

In this study in a similar land, among similar speaking people, different genetical background was detected. In the previous studies of Y-chromosome haplogroups of Iranian Arabs of Khuzestan Province, the highest frequency was observed for haplogroup F*(M89), followed by haplogroups J2*(M172), R1a1*(M17) and DE*(YAP) (2). Consistently, in our study haplogroup F-M89 (83.3%) and its subclads of J2-M172 (33.3%), and K-M9 (33.3%) showed higher frequencies. In a nearly comprehensive study on 15 Iranian ethnic groups, Y-chromosome bi-allelic markers were analyzed in 14 Iranian provinces containing Khuzestan and Isfahan (Chaharmahal and Bakhtiari was not included) (13). They reported J (31.4%) and R (29.1%) as the most frequent haplogroups that are in agreement with our results (33.3% of subjects for both J and K (as ancestor haplogroup of R)). J1-M267 is a less frequent subclad of J haplogroup in the majority of the Iranian samples (less than 10%) but reaches to 33.4% in South-Western part of Iran (Khuzistan Province) because of vicinity to Arab countries especially south of Iraq as involving the highest frequency of J1-M267 (13). However, it was not detected in our studied Arab subjects in Chaharmahal and Bakhtiari Province. J2-M172 is the main Iranian haplogroup (22.5%) but is less frequent in Arab countries as well as South and Southwestern provinces, for example, one case in Khuzestan (1.8%) (13). In our study J2-M172 was seen in two out of six subpopulations which reveals high frequency in comparison with later study. Moreover, observation of the high frequency of haplogroup J2 is inconsistent with its low frequency in neighboring countries of Saudi Arabia (J2:14%) and Iraq (13,15). On the other hand, high frequency of haplogroup K-M9 in this study is in contrast to the low frequency of this haplogroup observed in Saudi Arabia (about 5%) (16) as well as low frequency of subclads of haplogroup K such as haplogroup R in its southern neighbor provinces such as for example Khuzestan (13).

Generally, there were at least three genetically distinct ancestors with a possible time of origin of about 22200 yr before present (YBP) for haplogroup I-M170 (9), 42900 YBP for J-M304 (10) and 47400 YBP for K-M9 (11). As the most recent common ancestor (MRCA) of most these subpopulations, haplogroup F, lived very long years ago (about 40000–50000 YBP) (12) the data support nearly no close genetic relationship among most of these subpopulations except for subpopulations with same haplogroups of J2-M172 (n=2) and K-M9 (n=2). In this study various genetic backgrounds of Arab subpopulations living in this geographically conserved part of Iranian plateau are unexpected. Furthermore, presence of haplogroup I a predominantly European haplogroup considered as the only native European haplogroup (17, 18), in this province and among Arab subpopulations was not expected.

## Conclusion

Among observed haplogroups in these six paternally conserved but not-Semitic speaking Arab subpopulations in Chaharmahal and Bakhtiari Province, diverse haplogroups have been observed which are less consistent with haplogroup of neighboring Arab countries especially in haplogroup of K and I but in more agreement with haplogroups of Iranian Arabs especially in haplogroup of J2-M172.

## Ethical considerations

Ethical issues (Including plagiarism, informed consent, misconduct, data fabrication and/or falsification, double publication and/or submission, redundancy, etc.) have been completely observed by the authors.
